# Identification of a plasma proteomic signature associated with sudden cardiac death risk in the UK biobank

**DOI:** 10.3389/fcvm.2026.1831086

**Published:** 2026-05-08

**Authors:** Sijia Dai, Minjia Wu, Lei Zheng, Yuyu Chen, Shaoni Huang, Jiangyan Hao, Feiling Liu, Xiaowei He, Guangfeng Long, Yunfeng Zou, Cheng Xu

**Affiliations:** 1 Department of Toxicology, School of Public Health, Guangxi Medical University, Nanning, China; 2 Department of Neurosurgery, Children’s Hospital of Nanjing Medical University, Nanjing, China; 3 Editorial Office of Journal of Guangxi Medical University, Guangxi Medical University, Nanning, China; 4 Department of Clinical Laboratory, Children’s Hospital of Nanjing Medical University, Nanjing, China; 5 Guangxi Key Laboratory of Precision Medicine in Cardio-cerebrovascular Diseases Control and Prevention, Guangxi Medical University, Nanning, China; 6 Guangxi Clinical Research Center for Cardio-cerebrovascular Diseases, Guangxi Medical University, Nanning, China

**Keywords:** gene ontology analysis, plasma proteomics, proteomic signature, risk prediction, sudden cardiac death

## Abstract

**Background:**

Sudden cardiac death (SCD) remains difficult to predict in the general population, and proteomics may offer additional prognostic information. We aimed to develop and evaluate a plasma proteomic signature for SCD risk prediction in the UK Biobank.

**Methods:**

We analyzed prospective data from 52,826 UK Biobank participants, including 408 incident SCD cases. A proteomic signature was derived using repeated LASSO-Cox regression. Its biological relevance was explored by GO enrichment and PPI analyses. Predictive performance was assessed by discrimination, reclassification, calibration, and decision curve analysis.

**Results:**

A 71-protein signature was identified. Each 1-SD increase in the proteomic signature was associated with a higher risk of incident SCD (HR 3.25, 95% CI 2.94–3.58). Although non-proportional hazards were detected, the time-dependent association remained positive throughout follow-up. The proteomic-signature model showed substantially better discrimination than the clinical model (AUC 0.871 vs. 0.770), whereas the combined model provided only minimal additional improvement (AUC 0.872). Compared with the clinical model alone, the combined model improved reclassification (IDI 0.033, 95% CI 0.022–0.050; continuous NRI 0.403, 95% CI 0.351–0.459; both *p* < 0.001). Calibration was good, and net clinical benefit was mainly observed at relatively low threshold probabilities. Sensitivity analyses supported the robustness of the findings.

**Conclusion:**

The proteomic signature was independently associated with incident SCD and improved risk stratification beyond the clinical model. However, its clinical utility should be interpreted cautiously, and external validation is required before clinical application.

## Graphical Abstract

**Figure:**
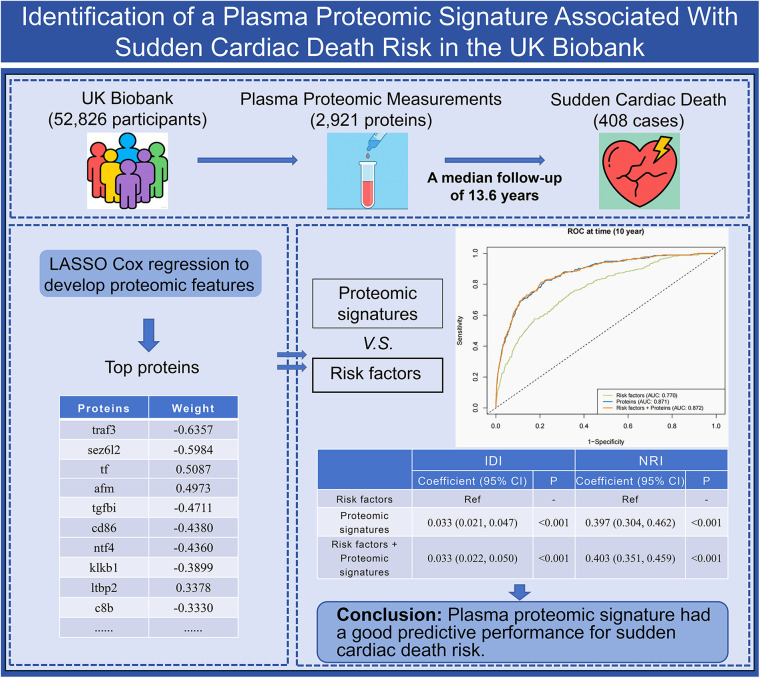
Using prospective data from 52,826 UK Biobank participants, we analysed 2,921 plasma proteins and identified 408 incident cases of sudden cardiac death (SCD) over a median follow-up of 13.6 years. A 71-protein plasma proteomic signature was derived using repeated LASSO-Cox regression. Compared with the conventional clinical risk-factor model, the proteomic signature demonstrated superior predictive performance and improved risk reclassification for SCD, highlighting the potential value of plasma proteomics for risk stratification in the general population.

## 1 Introduction

Sudden Cardiac Death (SCD) is defined as an unexpected death without a clear extracardiac cause. In witnessed cases, it is marked by a quick loss of consciousness, while in unwitnessed instances, it is defined as death within one hour of symptom onset. Despite significant progress in cardiovascular medicine in recent years, SCD still poses substantial medical and social challenges. According to the Lancet SCD Committee report, the number of SCD cases worldwide is around 4–5 million annually, making up more than 50% of all cardiovascular disease deaths (1). It seriously impacts public health and quality of life due to the sudden and deadly nature of SCD (2). Under current medical conditions, the chances of survival after SCD occurrence are very low. Therefore, early identification of individuals who may experience SCD is critical. Because many individuals did not meet the traditional criteria for enhanced monitoring or preventive intervention before the event occurred, the existing risk stratification strategies mainly rely on clinical characteristics and structural heart disease indicators, but their ability to identify high-risk individuals in the general population is still limited (3–5). Therefore, identifying biomarkers that can improve the prediction of SCD has important clinical implications.

In recent years, proteomics detection and analysis technologies have undergone rapid development. High-throughput proteomics (such as the Olink (6) and SomaScan (7) platforms) can now detect thousands of plasma proteins, making it possible to use biomarkers for disease prediction. These breakthroughs have generated new ideas for disease prediction and diagnosis. Existing research has confirmed that proteomics models outperform traditional risk scores in predicting cardiovascular diseases, such as atherosclerotic cardiovascular diseases (acute myocardial infarction, ischemic stroke, and cardiovascular death) (8–10), but the evidence for SCD is still limited. The UK Biobank (UKB), one of the largest prospective population cohorts globally, has collected genomic and follow-up data from over 500,000 participants, along with proteomic signature data from approximately 50,000 of them. This provides a robust basis for developing and evaluating proteomic predictors of SCD.

Based on the proteomic signature data from the UK Biobank, our aim is to construct and evaluate a proteomic signature for predicting SCD. Specifically, we aim to determine whether the proteomic signature constructed from plasma proteins can further enhance the risk discrimination ability beyond traditional clinical factors; and whether the selected proteins are enriched in pathways related to SCD that are biologically reasonable. We also assess the robustness of the results through a pre-defined sensitivity analysis.

## 2 Methods

### 2.1 Participants

The data for this study were obtained from the UK Biobank (UKB), which is currently the largest prospective human genetic cohort biobank in the world. Researchers recruited over 500,000 volunteers aged 40–69 in the UK between 2006 and 2010. Various data were collected through electronic questionnaires, online interviews, and physical measurements (11). The funding for this project was provided by the Medical Research Council (MRC), the Wellcome Trust, the British Heart Foundation, Cancer Research UK (CRUK), and the National Institute for Health and Care Research (12). All participants signed informed consent forms before joining the study. The UKB cohort study has received ethical approval from the National Information Governance Board for Health and Social Care and the North West Multi-Center Research Ethics Committee.

**Figure 1:**
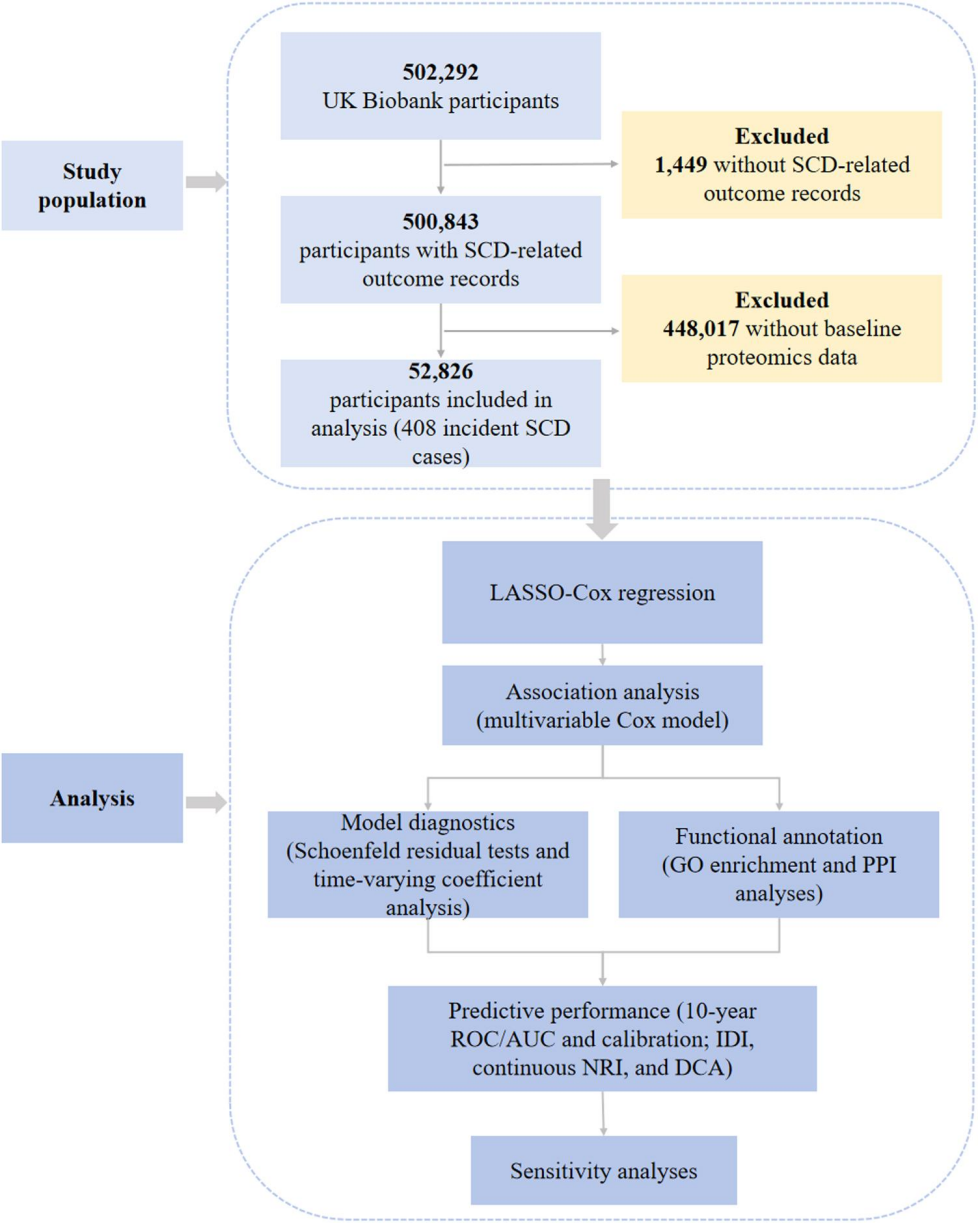
Flowchart of this study. The data for this study were obtained from the UK Biobank (UKB). According to the inclusion and exclusion criteria, a total of 52,826 participants were included in the analysis.

This study initially included a sample size of *n* = 502,292 from the UKB population. After excluding those without SCD-related information (*n* = 1,449), it was found that 500,843 participants had records of SCD-related outcomes. Additionally, participants with proteomics data in the baseline survey were selected, resulting in a final sample of 52,826 participants included in the analysis. The flowchart is shown in Figure 1.

### 2.2 Proteomics analysis

The UK Biobank Pharma Proteomics Project (UKB-PPP) Consortium performed proteomic analysis on blood samples collected during the baseline survey. Fasting plasma samples were collected in EDTA tubes at baseline, aliquoted, and stored at −80 °C to avoid loss of protein integrity due to repeated freeze–thaw cycles (13). Under strict quality control, the UKB-PPP Consortium used the Olink™ Explore 3,072 Proximity Extension Assay (PEA) to perform proteomic analysis on the plasma samples, detecting 2,941 protein analytes, including proteins from four panels covering cardiac metabolism, inflammation, neurology, and oncology. Ultimately, 2,923 characteristic proteins were identified (11). This study excluded proteins with a missing rate over 50%. Specifically, PCOLCE and NPM1 were removed at this step. Missing values in the remaining proteins were then imputed using the K-nearest neighbors (KNN) algorithm. In total, 2,921 proteins were included in the final analysis. Detailed information on the proteomics-related protein data is provided in Supplementary Table S1.

### 2.3 Determination of outcomes

The primary outcome of this study was incident SCD. According to the International Classification of Diseases, 10th Edition (ICD-10, https://biobank.ctsu.ox.ac.uk/crystal/field.cgi?id=41270), detailed descriptions and definitions are provided in Supplementary Table S2.

### 2.4 Covariates

Building on previous research on SCD risk factors (14), this study ultimately identified the covariates to include: age, gender, smoking status, systolic blood pressure (SBP), ethnicity, education level, baseline diabetes, use of antihypertensive drugs, blood albumin concentration, high-density lipoprotein cholesterol (HDL-C), and estimated glomerular filtration rate based on creatinine (eGFRcr). These were considered confounding factors between the exposure and the outcome in the study. Among them, age, SBP, albumin, HDL-C, and eGFRcr are continuous variables, while gender, smoking status, ethnicity, education level, diabetes, and use of antihypertensive drugs are categorical variables.

The demographic information, lifestyle factors, and disease history data were collected using a standardized questionnaire during the baseline survey. Smoking status was categorized as never, previous, current, or missing. SBP data were collected using an Omron digital blood pressure monitor (15). Ethnicity was classified as white, non-white, or missing. Baseline diabetes status was determined by whether the individual had been diagnosed with diabetes through self-report, was receiving treatment, had hospital diagnosis records, or had blood sugar levels (16). The information regarding the use of antihypertensive drugs was obtained from the questionnaire at the baseline survey. Serum albumin concentration was measured using a colorimetric method and a Beckman Coulter AU5800 assay (Beckman Coulter) (17). The blood HDL-C level was calculated using the enzyme-linked immunosorbent assay method on the Beckman Coulter AU5800 platform. The specific measurement procedures are available in the UK Biobank online resource center (https://biobank.ndph.ox.ac.uk/) (16). The eGFRcr level was calculated using the eGFRcr formula developed by the European Kidney Function Consortium, based on the creatinine value (18).

### 2.5 Statistical analysis

For the baseline characteristics between the SCD and non-SCD subjects, continuous variables were presented as mean ± standard deviation, and categorical variables were expressed as percentages, with missing values estimated using separate indicator categories. Before performing the least absolute shrinkage and selection operator (LASSO) regression analysis, for the missing values in the data, we employed the interpolation method based on the K-nearest neighbors (KNN) algorithm for processing. This method was implemented using the VIM package (version 6.6.2) by R software. And we reported missingness proportion per protein in Supplementary Table S3.

The plasma proteomic data of this study possess high-dimensional characteristics, and there are strong correlations among many proteins. Therefore, LASSO-Cox regression is more suitable for variable selection, dimension reduction, and reducing overfitting, while still being able to retain good model interpretability (11, 19). Protein features were screened using LASSO-penalized Cox proportional hazards regression. Because the plasma proteomic data were high-dimensional and highly correlated, this approach was used for variable selection and dimension reduction while retaining model interpretability. Before model fitting, protein levels were standardized to z scores. To improve selection stability, 10-fold cross-validation was repeated 100 times. In each repetition, the optimal penalty parameter (*λ*) was determined by cross-validation, and proteins with non-zero coefficients were recorded. Proteins selected in all 100 repetitions were defined as 100% reproducible proteins and were retained as the final signature proteins. The proteomic signature was calculated as the linear predictor from the final LASSO-Cox model, i.e., Proteomic signature = *Σβ*jZj, where *β*j denotes the final regression coefficient for protein j and Zj denotes the standardized value of protein j. For scoring new subjects, the same set of selected proteins and coefficients was applied, and each protein was standardized using the mean and standard deviation from the model-development dataset before calculating the weighted sum. The list of selected proteins and their coefficients is provided in Supplementary Table S4, and the corresponding R script for deriving the signature and scoring new subjects is provided in Supplementary Methods (Supplementary Code S1) (20). Then, a multivariate Cox proportional hazards model was used to estimate the hazard ratio (HR) and 95% confidence interval (95% CI) for the association between each additional standard deviation (SD) of proteomic signature and SCD. The model adjusted for age (continuous), gender (male/female), smoking status (never, previous, current), systolic blood pressure (SBP), ethnicity (white/non-white), education level, baseline diabetes status (yes/no), use of antihypertensive drugs (yes/no), blood albumin concentration, blood HDL-C levels, and estimated glomerular filtration rate using serum creatinine (eGFRcr). The proportional hazards assumption was assessed using Schoenfeld residuals with the cox.zph function in the survival package, and both the global test and the covariate-specific test for the proteomic signature were examined. When evidence of non-proportional hazards was observed, we further fitted a time-varying coefficient Cox model by including an interaction term between the proteomic signature and log(follow-up time). The time-dependent association was expressed as HR(t)=exp (*β*1+β2×log(t)). The corresponding Schoenfeld test results and time-dependent HR estimates are presented in Supplementary Tables S5, S6, and Supplementary Figure S1. GO analysis was performed on the selected proteins to identify the biological processes (BP), molecular functions (MF), and cellular components (CC) in which they are involved. Terms with a false discovery rate (FDR) <0.05 were considered significant (21). At the same time, protein-protein interaction (PPI) analysis was performed to calculate degree centrality, closeness centrality, and betweenness centrality, identifying key proteins. To assess whether proteomic signature can improve the prediction of SCD, we compared the predictive performance of three models: 1) SCD-related risk factors, 2) proteomic signatures, and 3) SCD-related risk factors with proteomic signatures. The discriminatory ability of the models was evaluated using the AUC. Further, the incremental predictive value brought by the proteomic signature was assessed using the integrated discrimination improvement (IDI) and the continuous net reclassification improvement (continuous NRI). In addition, calibration was evaluated by comparing predicted and observed 10-year survival probabilities using bootstrap resampling, and clinical utility was assessed using decision curve analysis across a range of threshold probabilities.

Finally, we conducted sensitivity analyses to evaluate the robustness of the observed associations to modeling choices and plausible unmeasured confounding. a) Exclude individuals diagnosed with SCD within one year after enrollment, b) Remove the education covariate. The inclusion and exclusion criteria for the study population, as well as all subsequent analyses, were performed using R software (version 4.1.1). Given the observational design, these analyses do not establish causality, and the findings should be interpreted as associations. The following packages were utilized: *glmnet*, *survival*, *clusterProfiler*, *timeROC*, and *survIDINRI*. Statistical significance was determined by a two-sided *p*-value < 0.05.

## 3 Results

### 3.1 Baseline characteristics

The study included 52,826 participants from the UK Biobank, with a mean age of 56.8 years (SD = 8.2); 46.1% were male. Among them, there were 408 incident SCD cases. Compared with the non-SCD population, SCD cases were older (61.6 vs. 56.8 years), had a higher proportion of males (65.7% vs. 45.9%), were more likely to smoke, had higher systolic blood pressure (143.1 vs. 139.5 mmHg), a greater proportion of patients with diabetes and those using antihypertensive drugs, and had lower albumin levels (44.3 vs. 45.1 g/L), lower HDL-C (1.3 vs. 1.4 mmol/L), and lower eGFRcr (77.3 vs. 86.0 ml/min/1.73 m²). There was no significant difference in ethnicity. Detailed data are shown in Table 1.

**Table 1:** Descriptive characteristics of participants in the UK biobank study based on the incidence of sudden cardiac death.

Characteristics	Incident SCD			
	Total (*N* = 52,826)	No (*N* = 52,418)	Yes (*N* = 408)	*P* value
Age (years, mean ± SD)	56.8 (8.2)	56.8 (8.2)	61.6 (7.0)	<0.001
Sex, male, *n* (%)	24,332 (46.1)	24,064 (45.9)	268 (65.7)	<0.001
Smoking status, *n* (%)				<0.001
Never	28,569 (54.1)	28,415 (54.2)	154 (37.8)	
Previous	18,435 (34.9)	18,256 (34.8)	179 (43.9)	
Current	5,568 (10.6)	5,494 (10.5)	74 (18.1)	
Missing value	254 (0.4)	253 (0.5)	1 (0.2)	
SBP (mmHg, mean ± SD)	139.6 (19.1)	139.5 (19.1)	143.1 (20.5)	<0.001
Ethnicity, *n* (%)				0.679
White	47,825 (90.5)	47,452 (90.5)	373 (91.4)	
Non-White	4,940 (9.4)	4,905 (9.4)	35 (8.6)	
Missing value	61 (0.1)	61 (0.1)	0 (0)	
Diabetes, *n* (%)				<0.001
Yes	3,002 (5.7)	2,934 (5.6)	68 (16.7)	
No	49,824 (94.3)	49,484 (94.4)	340 (83.3)	
Use of antihypertensive drugs, *n* (%)				<0.001
Yes	11,677 (22.1)	11,479 (21.9)	198 (48.5)	
No	41,149 (77.9)	40,939 (78.1)	210 (51.5)	
Albumin (g/L, mean ± SD)	45.1 (2.7)	45.1 (2.7)	44.3 (3.1)	<0.001
HDL-C (mmol/L, mean ± SD)	1.4 (0.4)	1.4 (0.4)	1.3 (0.4)	<0.001
eGFRcr (ml/min/1.73m², mean ± SD)	86.0 (13.6)	86.0 (13.5)	77.3 (18.5)	<0.001
Education, *n* (%)				<0.001
College or University degree	16,874 (31.9)	16,790 (32.0)	84 (20.6)	
A levels/AS levels or equivalent	5,782 (11.0)	5,753 (11.0)	29 (7.1)	
O levels/GCSEs or equivalent	10,907 (20.7)	10,816 (20.6)	91 (22.3)	
NVQ Or HND or HNC or equivalent	4,130 (7.7)	4,094 (7.8)	36 (8.8)	
Missing value	15,133 (28.7)	14,965 (28.6)	168 (41.2)	

SCD, sudden cardiac death; SBP, systolic blood pressure; HDL-C, high-density lipoprotein-cholesterol; eGFRcr, estimated glomerular filtration rate based on creatinine. Data are presented as means ± standard deviations (SDs), numbers, and percentages.

### 3.2 LASSO cox regression analysis of proteins and SCD

Using 100 repeated 10-fold cross-validated LASSO-Cox analyses, we identified 71 proteins that were selected in all repetitions and therefore considered 100% reproducible. These proteins were used to construct the proteomic signature. Among them, 44 proteins had positive coefficients and 27 had negative coefficients, with regression coefficients ranging from −0.64 to 0.51. The list of selected proteins, corresponding coefficients, and example R code for deriving the proteomic signature and scoring new subjects are provided in Supplementary Table S4 and Supplementary Methods (Supplementary Code S1). The association between the proteomic signature and SCD is shown in Supplementary Table S7. In the conventional Cox model, each 1-SD increase in the proteomic signature was associated with a higher average hazard of SCD over follow-up (HR 3.25, 95% CI: 2.94, 3.58).

### 3.3 Assessment of proportional hazards assumption and time-varying association

The proportional hazards assumption was assessed using Schoenfeld residuals. Evidence of non-proportionality was observed for the overall model (global test, *p* = 1.84 × 10^−4^) and for the proteomic signature term (covariate-specific test, *p* = 7.12 × 10^−6^) (Supplementary Table S5). We therefore performed a time-varying coefficient analysis by including an interaction term between the proteomic signature and log(follow-up time). The resulting time-dependent association was described as HR(t) = exp(1.5236630–0.1963018×log(t)), indicating that the magnitude of the association attenuated with longer follow-up. The estimated HRs were 4.589 at 1 year, 3.699 at 3 years, 3.345 at 5 years, and 2.919 at 10 years, while remaining above 1 throughout follow-up (Supplementary Table S6 and Supplementary Figure S1).

### 3.4 Enrichment and network analysis of candidate proteins

The enrichment analysis results showed that among the proteins in the proteomics signature, the most significantly enriched terms were negative regulation of response to external stimulus in GO biological process (BP) (Supplementary Figure S2, Supplementary Table S8), endopeptidase activity in GO molecular function (MF) (Supplementary Figure S3, Supplementary Table S9), and collagen-containing extracellular matrix in GO cellular component (CC) (Supplementary Figure S4, Supplementary Table S10). After performing GO analysis on the proteins in the proteomics signature, it was observed that in the PPI network, CXCL8 and EGFR showed the highest degree and betweenness centrality (Supplementary Table S11).

### 3.5 Discrimination, reclassification, calibration, and clinical utility assessment

**Figure 2:**
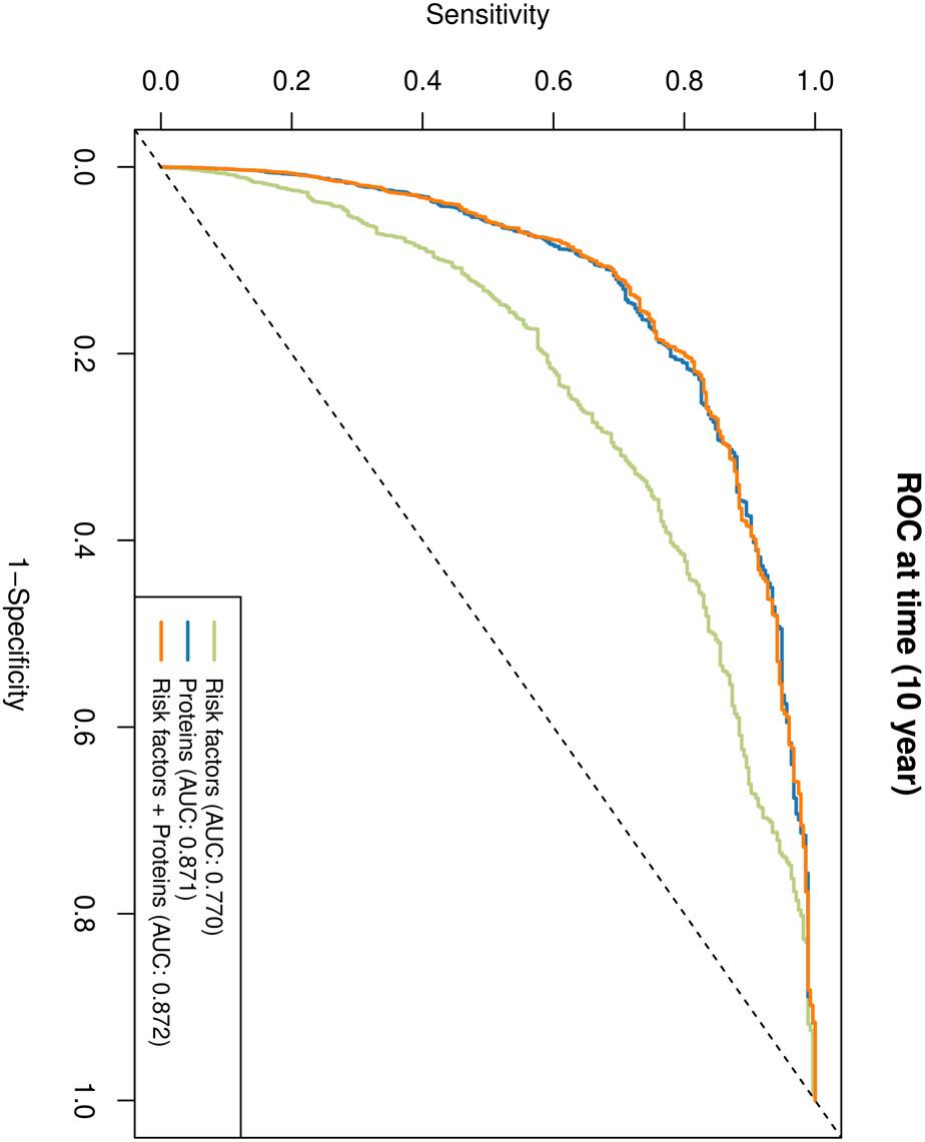
Receiver operating characteristic curves for various models in cohort analyses of 52,826 participants with 408 incident sudden cardiac death events. The AUC for SCD-related risk factors was 0.770, the proteomic-signature model had an AUC of 0.871, and the combined model (risk factors + proteomic signature) had an AUC of 0.872.

Figure 2 summarizes the predictive performance of the different models. The 10-year ROC analysis (Figure 2) showed that the AUC was 0.770 for the clinical risk-factor model, 0.871 for the proteomic-signature model alone, and 0.872 for the combined model. These results indicate that the proteomic-signature model substantially outperformed the clinical model, whereas adding clinical risk factors to the proteomic-signature model yielded only a minimal further increase in AUC. Table 2 summarizes the reclassification metrics of the proteomic-signature model and the combined model relative to the clinical model. In the key comparison of clinical relevance, the combined model improved reclassification over the clinical model alone (IDI 0.033, 95% CI 0.022–0.050; continuous NRI 0.403, 95% CI 0.351–0.459; both *p* < 0.001). Calibration analysis (Supplementary Figure S5) demonstrated good agreement between predicted and observed 10-year survival probabilities, with a mean absolute error of 0.005 and a 90th percentile absolute error of 0.01. Decision curve analysis (Supplementary Figure S6) further indicated that the combined model yielded net clinical benefit mainly within a relatively low threshold-probability range, whereas the added benefit became limited at higher thresholds.

**Table 2:** Incremental predictive value of proteomic signatures for SCD among study participants.

Model	IDI		Continuous NRI	
	Coefficient (95% CI)	*P*	Coefficient (95% CI)	*P*
Risk factors	Ref	–	Ref	–
Proteomic signatures	0.033 (0.021, 0.047)	<0.001	0.397 (0.304, 0.462)	<0.001
Risk factors + Proteomic signatures	0.033 (0.022, 0.050)	<0.001	0.403 (0.351, 0.459)	<0.001

CI, confidence interval; Ref, reference; SCD, sudden cardiac death; IDI, integrated discrimination improvement; NRI, continuous net reclassification improvement.

### 3.6 Sensitivity analysis

The results indicated that the hazard ratios remained materially unchanged across sensitivity analyses (3.15–3.25), with all *p*-values < 0.05 (Supplementary Table S12). These findings support the robustness of the association.

## 4 Discussion

In this prospective analysis of 52,826 UK Biobank participants, we identified a plasma proteomic signature associated with incident SCD and found that it provided incremental predictive value beyond established clinical risk factors. The proteomic-signature model showed substantially better discrimination than the clinical model, whereas adding clinical variables to the proteomic-signature model yielded only minimal additional improvement in AUC. By contrast, the combined model improved reclassification compared with the clinical model alone, suggesting that most predictive gain was driven by the proteomic signature itself. However, calibration analysis showed good agreement between predicted and observed 10-year survival probabilities, and decision curve analysis indicated that the combined model may offer clinical net benefit within a relatively low threshold-probability range. Functional analyses suggested that the selected proteins were mainly involved in inflammatory and extracellular matrix-related pathways, and the association between the proteomic signature and incident SCD remained robust in sensitivity analyses. Taken together, these findings suggest that the proteomic signature may add useful prognostic information, but its clinical utility should be interpreted cautiously and requires further validation. However, Schoenfeld residual testing indicated departure from the proportional hazards assumption, and time-varying coefficient analysis showed that the magnitude of the association attenuated with longer follow-up while remaining consistently positive throughout follow-up. Therefore, the HR from the conventional Cox model should be interpreted as an average association over the follow-up period rather than a constant effect at all time points. This pattern suggests that the proteomic signature may be particularly informative for earlier risk stratification. Together, these findings support the potential utility of proteomics for improving SCD risk stratification.

To better understand the biological relevance of the selected proteins, we performed GO enrichment and PPI network analyses on the proteins retained in the proteomic signature. Rather than indicating a single disease-specific pathway, the results suggest that the signature may reflect broader processes involved in cardiovascular injury, repair, and maladaptive remodeling (22–24). This interpretation is biologically plausible because multicellular remodeling programs involving immune activation, fibroblast responses, and tissue reorganization are increasingly recognized as key features of heart failure and other cardiovascular conditions associated with structural instability and adverse outcomes (22, 24). Although these findings do not establish causality, they support the biological plausibility of the proteomic signature identified in this study and are consistent with recent evidence that circulating proteomic signatures can yield mechanistic insights in common cardiac diseases (25).

The enrichment results further support this interpretation. In the current analysis, the top biological process terms were related to stimulus-response regulation and protein or peptide processing, whereas the leading molecular function and cellular component terms included endopeptidase activity and collagen-containing extracellular matrix, respectively; glycosaminoglycan binding also remained among the enriched molecular functions. These patterns suggest coordinated molecular responses to tissue stress together with extracellular matrix interaction and protease-associated remodeling, which are central features of cardiovascular injury and repair (23, 26, 27). In addition, extracellular matrix turnover, fibrosis, and inflammation-associated remodeling are highly relevant to cardiovascular disease because they can alter myocardial structure, promote electrical heterogeneity, and increase arrhythmogenic vulnerability (28, 29). Therefore, the identified proteomic signature may capture a biologically plausible axis linking tissue injury responses, matrix remodeling, and structural instability in cardiovascular disease (24, 28).

Our findings are consistent with a growing body of literature showing that large-scale plasma proteomics can improve disease prediction beyond conventional clinical variables. Prior studies have reported predictive value of proteomic models for cardiovascular outcomes such as ischemic stroke and broader cardiovascular events (30, 31). The present study extends this approach to SCD, a clinically important but difficult-to-predict outcome in the general population.

This study has several strengths. First, it was based on a large prospective cohort with well-characterized baseline data and long-term follow-up. Second, the use of high-throughput plasma proteomics enabled systematic evaluation of a broad range of candidate proteins. Third, we assessed both association and predictive performance and performed sensitivity analyses to examine the robustness of the findings. Nevertheless, these strengths should be interpreted in the context of several important limitations.

Several limitations should be acknowledged. First, the proteomics sub-cohort of the UK Biobank is not fully representative of the general population, and selection of participants into this sub-study may have introduced volunteer bias (32). As a result, the observed model performance may not directly generalize to higher-risk clinical populations. Second, incident SCD was identified from linked death records using ICD-10 codes rather than formal adjudication by cardiovascular specialists. Accordingly, we could not distinguish arrhythmic SCD from other cardiovascular deaths and lacked information on the circumstances of death, witness status, and resuscitation attempts, which may have led to outcome misclassification (33). Third, as in all high-throughput proteomics studies, measurement error related to platform characteristics, sample handling, and assay performance cannot be completely excluded. Fourth, the model was developed and evaluated within the same dataset, and although repeated cross-validation was used during feature selection, external validation is still required to assess generalizability and potential overfitting. Finally, this was an observational study, and the identified proteins should be interpreted as correlates of SCD risk rather than causal determinants. Age, gender and ethnicity all have an impact on the baseline levels of circulating proteins and the risk of sudden cardiac death (SCD). For instance, age-related changes in protein levels may alter the interpretation of protein group characteristics among different age groups. Ethnicity may also affect the expression of certain proteins due to genetic, environmental and lifestyle factors. Therefore, it is necessary to further validate our protein group characteristics in different populations and age groups to assess their general applicability and ensure the robustness of the prediction performance across different subgroups of the population. Future studies should focus on external validation of the proteomic signature in independent and more diverse populations, including higher-risk clinical cohorts. Improved outcome adjudication using linked clinical, emergency-response, and forensic information would strengthen the validity of SCD classification in future studies. In addition, mechanistic studies are needed to clarify whether hub proteins such as CXCL8 and EGFR are directly involved in biological pathways linked to SCD. Finally, in the future, it can be further explored whether other machine learning or ensemble methods can bring additional gains while maintaining biological interpretability.

## 5 Conclusion

In summary, we constructed a proteomic signature related to sudden cardiac death in the UK Biobank. The proteomic signature showed substantially better discrimination than the clinical risk-factor model and suggested biologically plausible pathways related to stimulus-response regulation, proteolytic activity, and extracellular matrix-associated components. However, although discrimination and reclassification improved compared with the clinical model alone, the additional discrimination gain of the combined model beyond the proteomic-signature model was minimal, and the model's clinical net benefit appeared to be concentrated within a limited threshold-probability range despite good internal calibration. Time-varying analyses further showed that the association attenuated over time but remained consistently adverse throughout follow-up. These findings support the potential utility of the proteomic signature for SCD risk stratification, but further external validation and assessment of temporal performance are needed before clinical application.

## Data Availability

The data analyzed in this study is subject to the following licenses/restrictions: The datasets analyzed in this study are not publicly available. Access to UK Biobank data is restricted and requires formal application and approval by the UK Biobank. This study was conducted under UK Biobank Application Number 140851. Requests to access these datasets should be directed to https://www.ukbiobank.ac.uk/. The R code used to derive the proteomic signature, identify the 100% reproducible proteins across 100 repeated 10-fold cross-validations, and score new subjects is provided in the Supplementary Materials as Code S1.
