# Supplementary figures and images for: Identification of a plasma proteomic signature associated with sudden cardiac death risk in the UK biobank
Source: Front Cardiovasc Med. 2026 May 8;13:1831086. doi: 10.3389/fcvm.2026.1831086 (PMC13193928; doi:10.3389/fcvm.2026.1831086)

Time-dependent HR for protein\_score\_sd

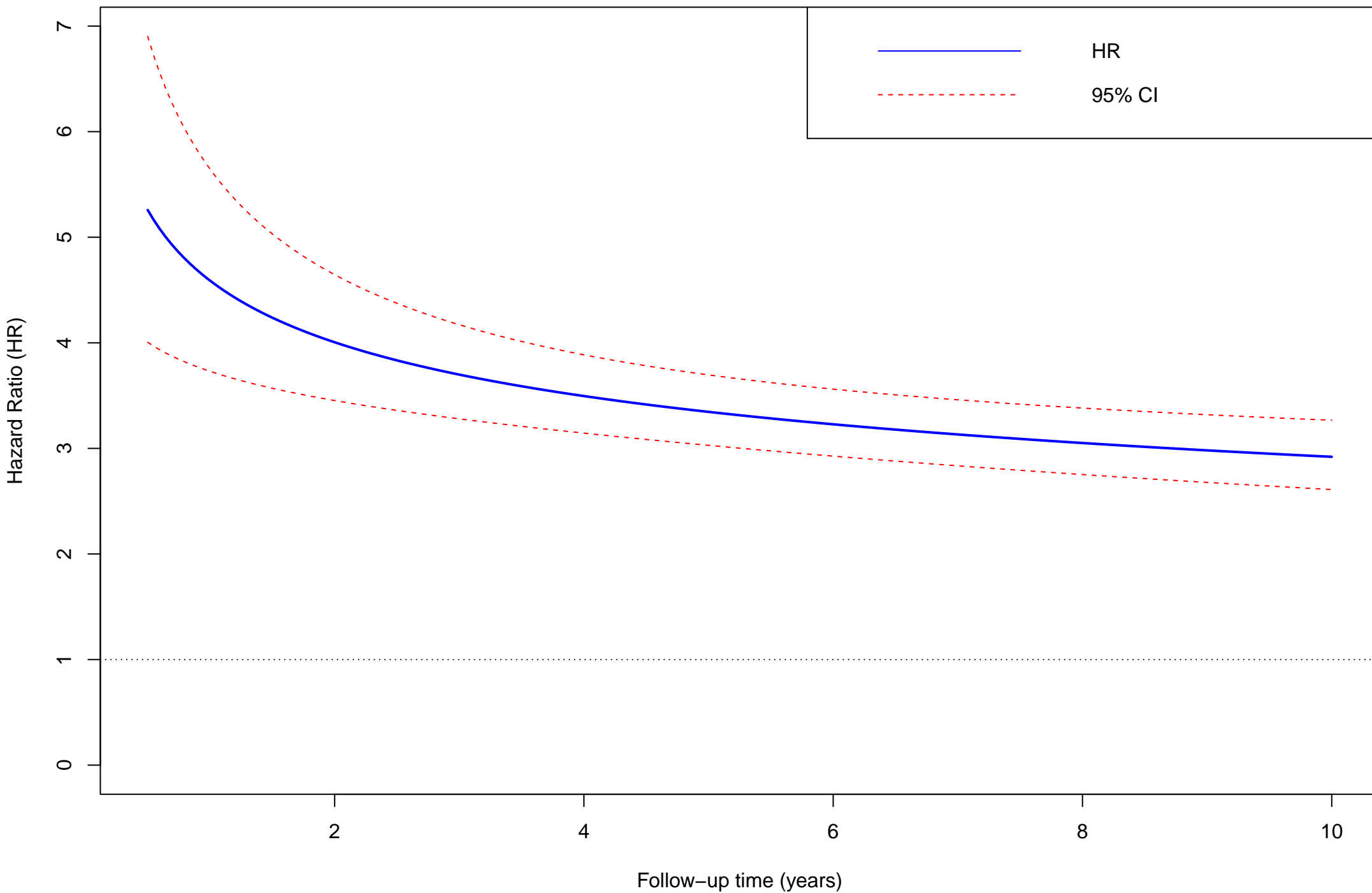

Supplement: Supplementary file 1 [file Image1.pdf]

# GO Biological Process (BP) Enrichment

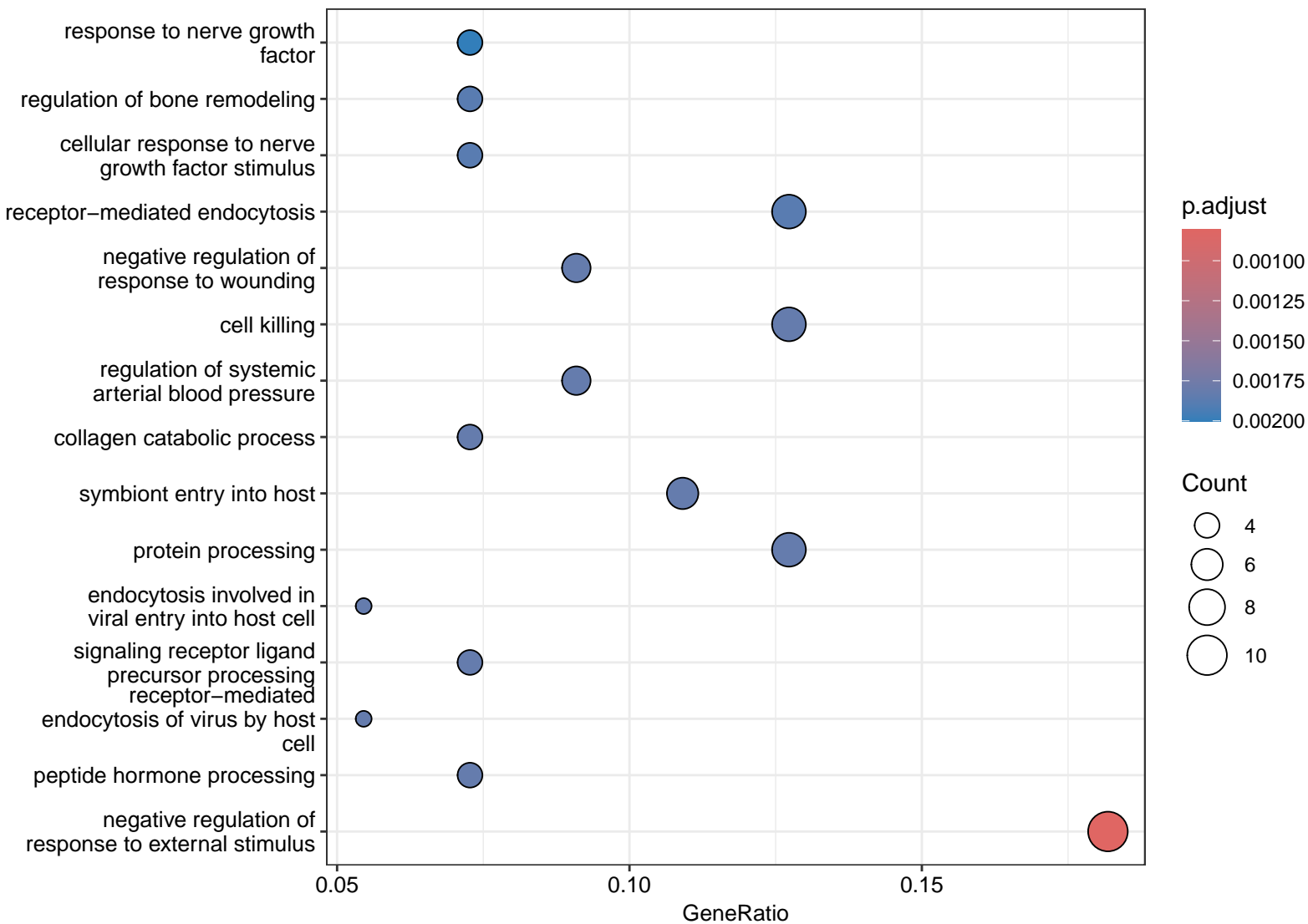

Supplement: Supplementary file 2 [file Image2.pdf]

# GO Molecular Function (MF) Enrichment

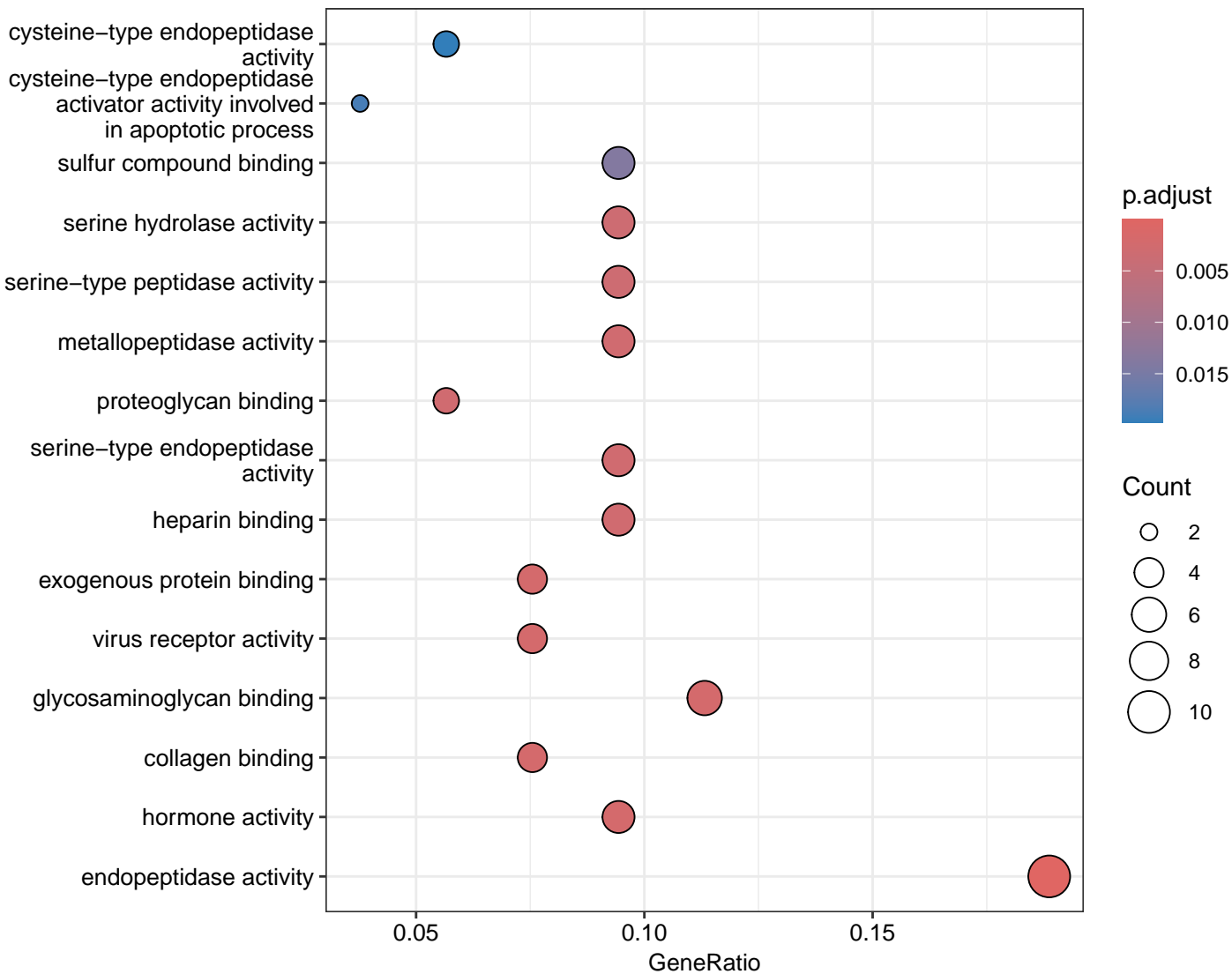

Supplement: Supplementary file 3 [file Image3.pdf]

# GO Cellular Component (CC) Enrichment

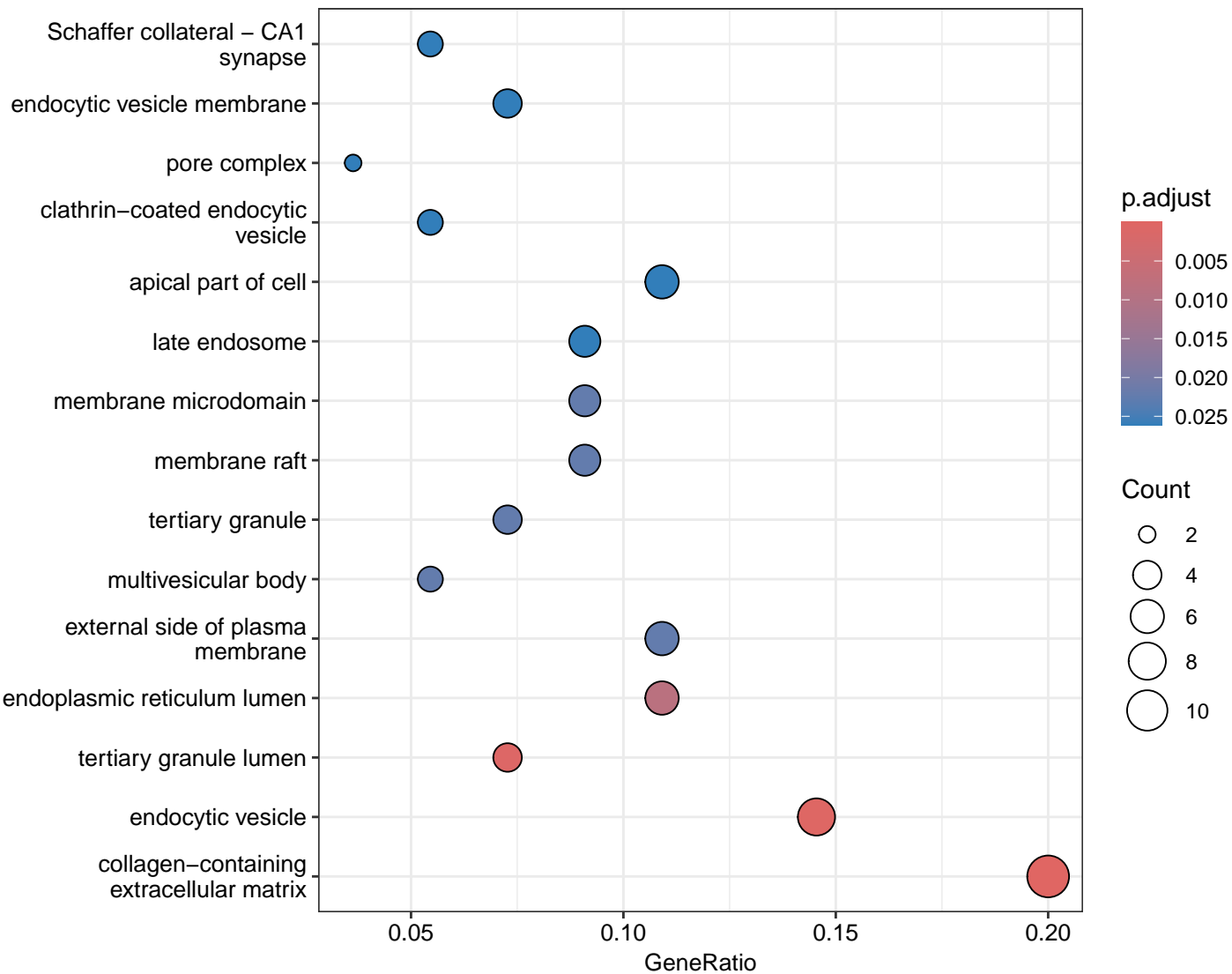

Supplement: Supplementary file 4 [file Image4.pdf]

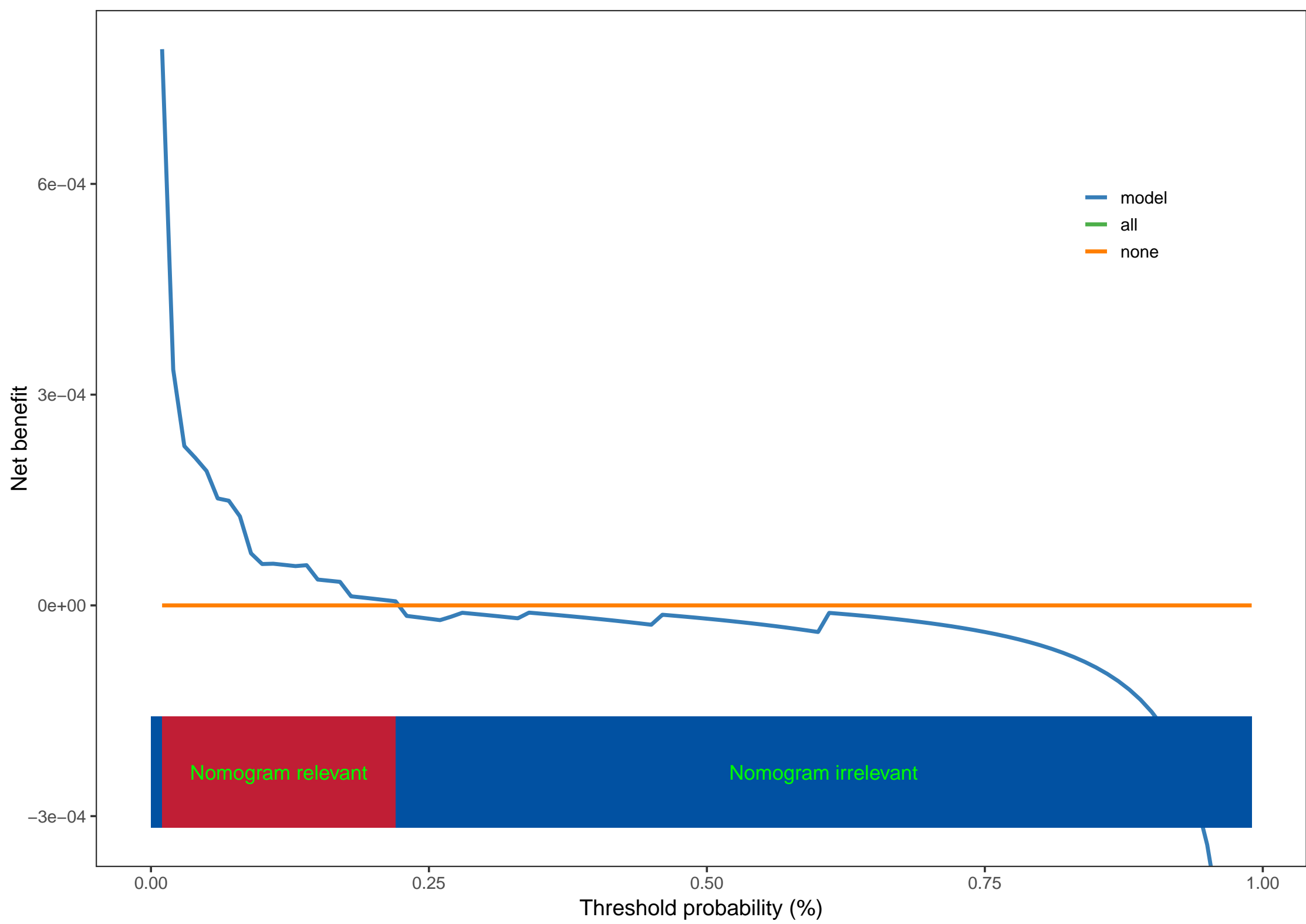

Supplement: Supplementary file 6 [file Image6.pdf]
