# Supplementary material for: Identification of a plasma proteomic signature associated with sudden cardiac death risk in the UK biobank
Source: Front Cardiovasc Med. 2026 May 8;13:1831086. doi: 10.3389/fcvm.2026.1831086 (PMC13193928; doi:10.3389/fcvm.2026.1831086)
Supplement: Supplementary file 5 [file Image5.pdf]

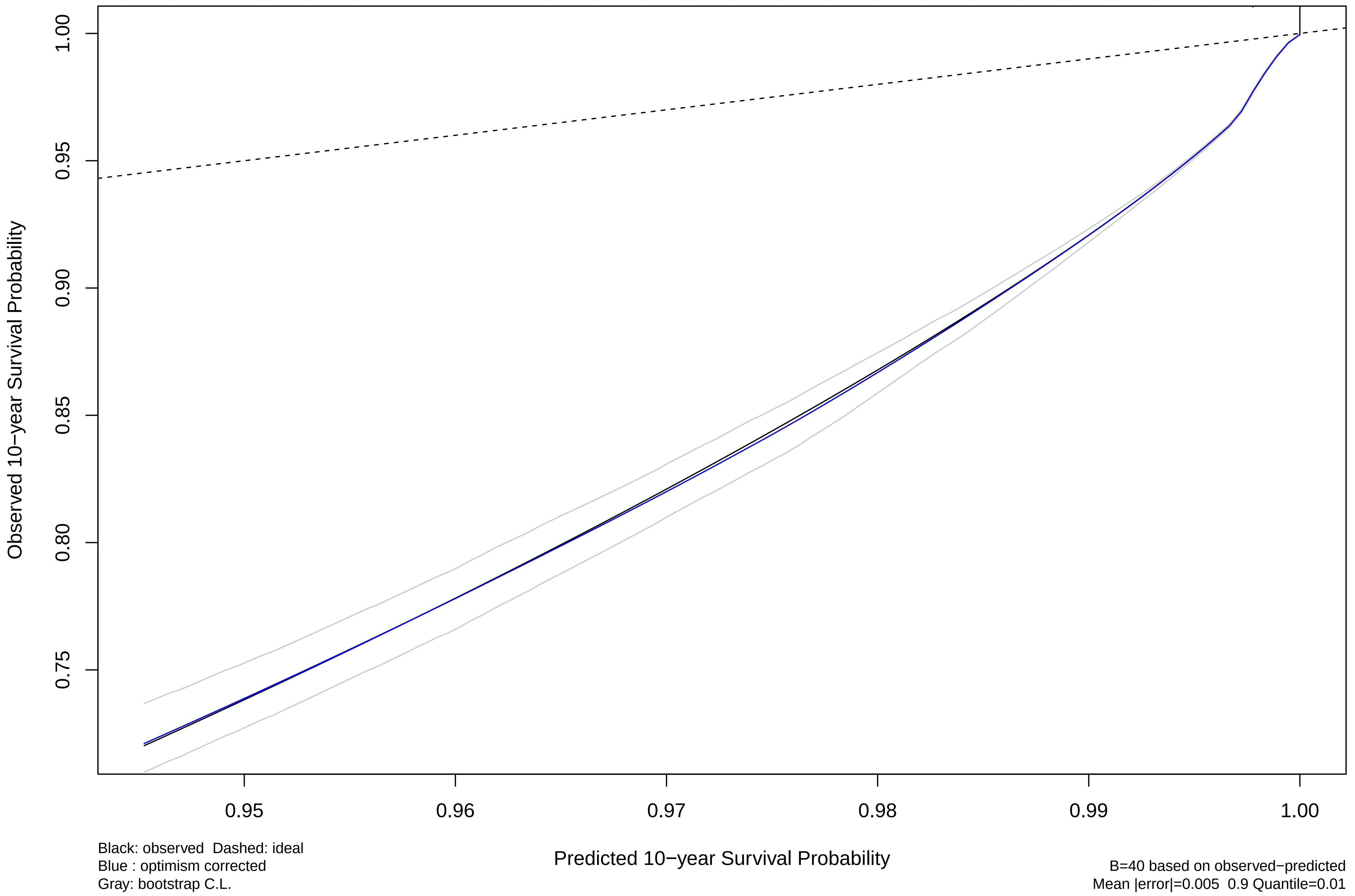

Observed 10−year Survival Probability
0.75
0.80
0.85
0.90
0.95
1.00
Predicted 10−year Survival Probability
0.95
0.96
0.97
0.98
0.99
1.00
Black: observed  Dashed: ideal
Blue : optimism corrected
Gray: bootstrap C.L.
B=40 based on observed−predicted
Mean |error|=0.005  0.9 Quantile=0.01
