# Supplementary material for: Identification of a plasma proteomic signature associated with sudden cardiac death risk in the UK biobank
Source: Front Cardiovasc Med. 2026 May 8;13:1831086. doi: 10.3389/fcvm.2026.1831086 (PMC13193928; doi:10.3389/fcvm.2026.1831086)
Supplement: Supplementary file 8 [file Table2.docx]

setwd("D:/带教学生/代四佳/sudden death")

library(haven)

library(survival)

library(glmnet)

library(dplyr)

library(doSNOW)

# =========================

# 0. 读取数据

# =========================

dat <- read_dta("蛋白KNN填补_最终版.dta")

dat <- dat[dat$SCD_time_follow_time > 0, ]

y <- Surv(dat$SCD_time_follow_time, dat$SCD)

protein_index <- c(2:8, 16, 17, 19, 27:2945)

x_all <- as.matrix(dat[, protein_index])

storage.mode(x_all) <- "double"

# 剔除方差为零的变量（加速并避免警告）

zero_var <- apply(x_all, 2, var) == 0

x_all <- x_all[, !zero_var]

cat("剔除零方差变量后剩余变量数:", ncol(x_all), "\n")

# =========================

# =========================

# 1. 重复 20 次 10 折 CV LASSO-Cox（稳定蛋白筛选）

# =========================

n_rep <- 20 # 原为100，改为20次足够

selected_list <- vector("list", n_rep)

for (i in seq_len(n_rep)) {

set.seed(2025 + i)

foldid_i <- sample(rep(1:10, length.out = nrow(x_all)))

cv_fit_i <- cv.glmnet(

x = x_all, y = y, family = "cox", alpha = 1,

nfolds = 10, foldid = foldid_i, type.measure = "C",

standardize = TRUE, parallel = TRUE,

nlambda = 30, # 减少 lambda 数量

thresh = 1e-4, # 放宽收敛精度

maxit = 500 # 限制最大迭代

)

coef_i <- as.matrix(coef(cv_fit_i, s = "lambda.min"))

selected_list[[i]] <- rownames(coef_i)[coef_i[, 1] != 0]

cat("完成第", i, "/", n_rep, "次重复\n")

}

# 统计选择频率

selection_freq <- sort(table(unlist(selected_list)), decreasing = TRUE)

selection_df <- data.frame(

Proteins = names(selection_freq),

Selection_frequency = as.integer(selection_freq),

stringsAsFactors = FALSE

)

# 提取 20 次重复中 100% 被选中的稳定蛋白

stable_proteins <- selection_df$Proteins[selection_df$Selection_frequency == n_rep]

# =========================

# 2. 基于稳定蛋白的最终 LASSO-Cox 模型

# =========================

if (length(stable_proteins) > 0) {

x_stable <- as.matrix(dat[, stable_proteins, drop = FALSE])

storage.mode(x_stable) <- "double"

set.seed(3025)

foldid_final <- sample(rep(1:10, length.out = nrow(x_stable)))

cv_fit_final <- cv.glmnet(

x = x_stable, y = y, family = "cox", alpha = 1,

nfolds = 10, foldid = foldid_final, type.measure = "C",

standardize = TRUE, parallel = TRUE,

nlambda = 30, thresh = 1e-4

)

final_model <- glmnet(

x = x_stable, y = y, family = "cox", alpha = 1,

lambda = cv_fit_final$lambda.min, standardize = TRUE

)

coef_final <- as.matrix(coef(final_model))

table_s3 <- data.frame(

Proteins = rownames(coef_final),

Coefficients = as.numeric(coef_final[, 1]),

stringsAsFactors = FALSE

) %>%

left_join(selection_df, by = "Proteins") %>%

filter(Coefficients != 0) %>%

arrange(Coefficients)

write.csv(table_s3, "Table_S3_LASSO_coefficients.csv", row.names = FALSE, quote = FALSE)

cat("最终模型系数表已保存为 Table_S3_LASSO_coefficients.csv\n")

} else {

cat("警告：未找到任何在20次重复中全部被选中的稳定蛋白。\n")

}
